# Alterations in LDL and HDL after an ischemic stroke associated with carotid atherosclerosis are reversed after 1 year‡

**DOI:** 10.1016/j.jlr.2024.100739

**Published:** 2024-12-31

**Authors:** Núria Puig, Pol Camps-Renom, Martin Hermansson, Ana Aguilera-Simón, Rebeca Marín, Olga Bautista, Noemi Rotllan, Nerea Blanco-Sanroman, Maria Constanza Domine, Katariina Öörni, José Luis Sánchez-Quesada, Sonia Benitez

**Affiliations:** 1 Cardiovascular Biochemistry Group, Institut de Recerca Sant Pau, (IR Sant Pau), Barcelona, Spain; 2 Stroke Unit, Department of Neurology, Hospital de La Santa Creu I Sant Pau, IR Sant Pau, Barcelona, Spain; 3 Atherosclerosis Research Laboratory, Wihuri Research Institute, Helsinki, Finland; 4 Pathophysiology of Lipid-Related Diseases, Research Institute Sant Pau (Institut de Recerca Sant Pau, IR Sant Pau), Barcelona, Spain; 5 CIBER of Diabetes and Metabolic Diseases (CIBERDEM), Madrid, Spain; 6 Department of Neurology, Hospital de La Santa Creu I Sant Pau, Barcelona, Spain

**Keywords:** lipoproteins, LDL oxidation, atherosclerosis, apolipoproteins, ceramides, inflammation, ischemic stroke, carotid plaque, LDL aggregation, HDL

## Abstract

Approximately, 20% of ischemic strokes are attributed to the presence of atherosclerosis. Lipoproteins play a crucial role in the development of atherosclerosis, with LDL promoting atherogenesis and HDL inhibiting it. Therefore, both their concentrations and their biological properties are decisive factors in atherosclerotic processes. In this study, we examined the qualitative properties of lipoproteins in ischemic stroke patients with carotid atherosclerosis. Lipoproteins were isolated from the blood of healthy controls (n = 27) and patients with carotid atherosclerosis (n = 64) at 7 days and 1 year postischemic stroke. Compared to controls, patients’ LDL 7 days poststroke showed increased levels of apoC-III, triacylglycerol, and ceramide, along with decreased cholesterol and phospholipid content. LDL from patients induced more inflammation in macrophages than did LDL from controls. HDL isolated from patients 7 days after stroke showed alterations in the apolipoprotein cargo, with reduced levels of apoA-I and increased levels of apoA-II, and apoC-III compared to controls. Patients’ HDL also showed a higher electronegative charge than that of controls and partially lost its ability to counteract the modification of LDL and the inflammatory effects of modified LDL. One year after stroke onset, the composition of patients’ LDL and HDL resembled those of the controls. In parallel, LDL and HDL gained positive charge, LDL became less prone to oxidation and aggregation, and HDL regained protective properties. In conclusion, LDL and HDL in ischemic stroke patients with carotid atherosclerosis exhibited alterations in composition and function, which were partially reversed 1 year after stroke.

## Graphical abstract

**Figure:**
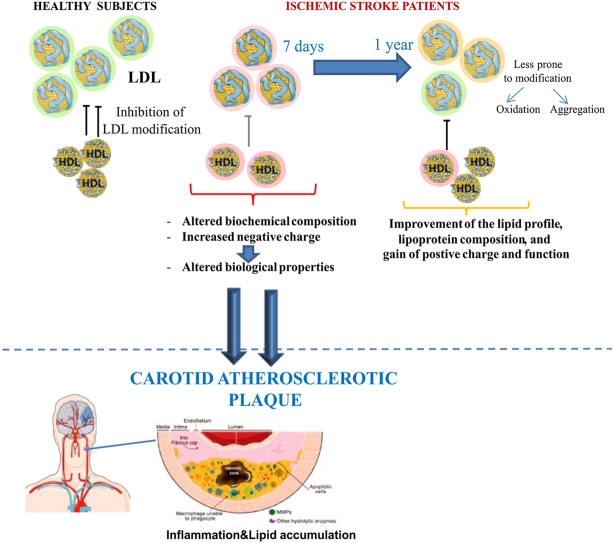

Ischemic stroke is a leading cause of death and disability worldwide (1, 2). The presence of atherosclerotic plaques is the cause of approximately 20% of all ischemic strokes, specifically named atherothrombotic strokes. This type of stroke carries a higher risk of major vascular events and stroke recurrence compared to other stroke subtypes (3). Atherosclerotic plaques are predominantly located in the internal carotid artery (ICA). Carotid endarterectomy—a surgical procedure to remove these plaques—is a key strategy for secondary prevention of this stroke subtype, and its implementation is primarily based on the degree of stenosis (4). However, the vulnerability of carotid plaques is greatly influenced by their inflammatory state and lipid content (5). Lipoproteins play a key role in atherosclerosis. LDL, particularly when modified by oxidation and/or aggregation in the arterial wall, triggers inflammation and lipid accumulation, leading to the formation and progression of atherosclerotic lesions (6). Conversely, HDL is considered an atheroprotective lipoprotein that can inhibit LDL modification and reverse some of the atherogenic effects of modified LDL (7).

Numerous epidemiological studies have examined the relationship between lipid profiles and ischemic stroke. High levels of plasma total cholesterol (TC), particularly LDL-C, are associated with an increased incidence of atherothrombotic stroke (8, 9) and the progression of carotid stenosis (10). Hence, lowering TC and LDL-C levels through therapy prevents the progression of carotid atherosclerosis in asymptomatic patients (11) and ischemic stroke patients (12). For these patients, lipid-lowering therapy also reduces the risk of recurrence and future cardiovascular (CV) events (13). However, HDL-C levels are known to have a strong inverse association with coronary (14) and atherothrombotic stroke (15, 16), although the usefulness of HDL-C as a biomarker for stroke has been questioned (17).

There is growing evidence that lipoprotein concentrations are not always the primary pathogenic factor in atherosclerosis-related diseases. Therefore, the presence of atherosclerosis is not solely determined by high levels of LDL-C (18). Patients with CV conditions, such as type 2 diabetes, obesity, or metabolic syndrome, may exhibit normal TC and LDL-C levels but have altered qualitative lipoprotein properties that favor the development of subclinical atherosclerosis (19).

Recently, we showed that ischemic stroke patients with carotid atherosclerosis, despite having normal lipid profiles, exhibited higher proportion of a modified LDL, named electronegative LDL (LDL(−)), in blood (20). LDL(−) constitutes about 3–5% of the total LDL in healthy subjects, has negative charge, increased susceptibility to aggregation, and inflammatory properties (21). The proportion of LDL(−) is increased in pathologies associated with CV risk (22, 23) and in the presence of subclinical atherosclerosis, where it correlates with the extent of carotid stenosis (24). LDL(−) levels increase during the acute phase of myocardial infarction (25) and after ischemic stroke (20, 26). In this context, Shen *et al.* suggested that LDL(−) may serve as a marker of plaque vulnerability in ischemic stroke patients (26). Interestingly, in our cohort of patients, LDL(−) levels were associated with some features of carotid plaque vulnerability (20).

In addition to plasma lipoprotein levels, the concentrations of some of their components, such as apolipoproteins, including apoB, apoA-I, and apoC-III, has been associated with atherothrombotic stroke (27, 28, 29). However, the specific lipid and apolipoprotein composition of lipoproteins and their relationship with atherothrombotic stroke remain largely unknown. Some studies have reported differences in the particle size of HDL and LDL in ischemic stroke patients (30, 31), as well as alterations in lipid and protein composition in lipoproteins isolated from patients with carotid stenosis (32, 33, 34). Additionally, changes in the HDL proteome in ischemic stroke patients have been related to diminished protective function (30, 31, 35).

In our ischemic stroke cohort, the increased proportion of LDL(−) associated with carotid plaque vulnerability (20) suggested underlying alterations in the composition and biological properties of LDL. In addition, the crucial protective role of HDL against the atherogenicity of LDL(−) may also have been compromised in these patients. In the present study, we examined the qualitative properties of LDL and HDL 7 days and 1 year after the onset of stroke.

## Material and Methods

### Study design

We conducted an observational cohort study (NCT03218527) in the Hospital de la Santa Creu i Sant Pau (Barcelona, Spain) between January 2016 and March 2019. The study population comprised consecutive adult patients who had experienced recent anterior circulation ischemic strokes and carotid atherosclerosis. We also included a control group of healthy subjects. The hospital’s Ethics Committee approved the study (IIBSP-LRB-2017-54, June 26, 2017), and the participants gave their written informed consent to participate. The study was performed in accordance with the Declaration of Helsinki.

### Study population

The patients included in the study met the following criteria: 1) age ≥50 years; 2) anterior circulation ischemic stroke or transient ischemic attack (TIA) within 7 days before inclusion; 3) the presence of at least one atherosclerotic plaque in the ICA on the side consistent with stroke symptoms, regardless of the degree of stenosis. Carotid stenosis was graded with computed tomography or magnetic resonance imaging-angiography when available using the NASCET approach (36), and ultrasound based on hemodynamic criteria (37); and 4) a modified Rankin Scale score <4 before inclusion.

Exclusion criteria were as follows: 1) the presence of definitive cardioembolic, lacunar, or unusual stroke etiology according to the TOAST criteria (38); 2) the occurrence of a hemodynamic stroke/TIA; 3) previous carotid surgery or stenting; 4) the presence of comorbidities conditioning a life expectancy <1 year; 5) suspected concomitant infections at the time of blood extraction; and 6) total carotid artery occlusion.

Healthy volunteers included in the control group fulfilled the following criteria: 1) age ≥50 years, 2) no prior history of ischemic heart disease, and 3) no prior history of stroke. There were no clinical recommendation or lifestyle changes resulting from their participation in the study. As they were healthy volunteers with no recent history of stroke or other CV events, only a single point of blood collection was available for this group of subjects.

### Clinical and biochemical variables

The following variables were recorded for stroke patients: 1) age and sex; 2) past medical history including hypertension, diabetes, dyslipidemia, previous stroke/TIA, coronary artery disease, and tobacco and alcohol consumption; 3) previous drug treatment; 4) National Institutes of Health Stroke Scale score, as a surrogate of infarct size; 5) BMI, divided into three categories (healthy weight, BMI 18.5 to < 25; overweight, BMI 25 to < 30; and obese, BMI ≥30); 6) regular physical exercise according to the Physician-Based Assessment and Counseling for Exercise scale (39); 7) Mediterranean diet adherence according to the PREDIMED score (40); 8) modified Rankin Scale score at inclusion; 9) stroke etiology according to the TOAST criteria (38) after a diagnostic work-up that included at least a 24-h-electrocardiogram, an echocardiogram, and an ultrasound carotid examination; and 10) results of an admission blood test including renal function, hemogram, hemostasis, and lipid profile analysis.

All participants in the control group underwent clinical interviews to assess their demographics, lifestyle habits, and previous drug treatments. Additionally, carotid ultrasound examinations were performed to rule out the presence of asymptomatic high-degree stenosis in both ICA and an electrocardiogram to rule out silent ischemic heart disease and atrial fibrillation.

### Sample collection

Peripheral blood samples were collected from the stroke patients 7 days and 1 year after stroke (labeled 7 days and 1 year in the figures). Peripheral blood was also collected from healthy controls (labeled Ctr in the figures). Patients and controls were in fasting conditions before blood extraction. Plasma was collected in EDTA-containing Vacutainer tubes, and serum was collected in Serum Separator Tubes coated with a clot activator. The tubes were centrifuged at 1,500 *g* for 15 min at 4°C, and aliquots were frozen at −80°C for up to 4 years until analysis.

### Serum determinations

Serum lipid profiles, including triacylglycerol (TAG), TC, LDL-C, and HDL-C, were measured using commercial standardized methods (Alinity ci-series, Abbott Core Laboratory, Chicago, IL). ApoA-I and apoB were measured using a Cobas c501 autoanalyzer (Roche Diagnostics, Basel, Switzerland). ApoJ levels in the serum were evaluated using a commercial ELISA kit (Mabtech, Stockholm, Sweden). LDL particle size and HDL subfraction proportions in the serum were assessed using nondenaturing polyacrylamide gradient (2.5–16%) gel electrophoresis (GGE), as previously described (41). Briefly, lipoproteins were prestained with the lipophilic dye Sudan Black. LDL size was measured using an LDL size standard, consisting of a plasma mixture with LDL particles of varying sizes (39). The relative proportion of HDL2 and HDL3 was quantified according to the density of the bands corresponding to each HDL fraction (19).

### Lipoprotein isolation and composition

Lipoproteins were isolated from 1 ml of plasma (1.006 g/ml) taken from stroke patients and healthy controls using flotation sequential ultracentrifugation according to the density of the lipoprotein: VLDL (<1.019 g/ml), LDL (1.019–1.063 g/ml), and HDL (1.063–1.210 g/ml) by adding KBr (42). Samples were centrifuged for a minimum of 20 h at 36,000 rpm, and 8°C with a Kontron TFT 45.6 (max speed: 45,000 rpm; max RCF: 238,859 g (Outer Row); 204,252 g (Inner Row)) in the Beckman Optimal 90 K Ultracentrifuge, to isolate the lipoproteins.

All analyses related to lipoprotein subfractions were performed within 5 days of isolation, except for the lipidomic analysis, where LDL was frozen for 2 months before the lipid extraction.

The lipid and apolipoprotein composition of the isolated lipoproteins was determined by measuring the content of TC, TAG, apoB, apoA-I (Roche Diagnostics, Basel, Switzerland), phospholipids (PLs), free cholesterol (FC) (FUJIFILM Wako Chemicals, Osaka, Japan), apoA-II, apoE, and apoC-III (Kamiya Biomedicals, Seattle, Washington) using a Cobas c501 autoanalyzer. ApoJ levels were evaluated using commercial ELISA (Mabtech, Stockholm, Sweden).

### Mass-spectrometric analysis of LDL lipid composition

LDL lipid compositions were determined using shotgun lipidomics, that is, direct infusion MS.

Sample input was normalized by diluting 10 μg isolated LDL (per total protein basis) in 200 μl of 200 mM ammonium formate and spiked with 10 μl of a quantitative standard cocktail (Splash Lipidomix, Avanti, Alabaster, AL), which provided a single internal standard (IS) for each targeted lipid class (listed in Supplemental Table S1). The total lipids were extracted in silylated 2 ml screw-cap vials following a scaled-down version of the Bligh and Dyer protocol (43), modified by substituting NaCl with 200 mM ammonium formate. The lipid-containing lower phases were collected, vacuum-evaporated, and the residual lipids were immediately dissolved in 200 μl of chloroform/methanol (1:2, v/v) before storage at −20°C pending analysis.

Immediately prior to MS analysis, the reconstituted lipids were mixed 1:1 (vol/vol) with 1.33 mM ammonium formate dissolved in 2-propanol and infused into the ESI source the mass spectrometer using a Hamilton micro syringe pump set to a flow rate of 8 μl/min.

Mass spectrometric analyses were conducted using an Agilent 6,410 Triple Quadrupole mass spectrometer equipped with an ESI source (Agilent Technologies, Palo Alto, CA). Data acquisition was managed through Agilent MassHunter Workstation Acquisition software (version B.01.03, Agilent Technologies, Palo Alto, CA). The instrument operated in both positive and negative ionization modes, allowing for selective detection across various lipid classes. Ionization and fragmentation conditions were optimized individually for each lipid class (detailed in Supplemental Table S2).

The typical parameters were as follows: gas temperature of 250°C, capillary voltage 5,000 V, and nebulizer pressure (using nitrogen gas) at 40 psi. Collision energy settings ranged from 13 to 48 V, while the fragmentor voltage was adjusted between 75 and 380 V, and the cell accelerator was set between 4 and 5 V. The dwell time was (scan rate) was set between 2000 and 2,800 ms. Mass resolutions for the MS1 and MS2 quadrupoles, defined by the peak full-width at half-maximum, were maintained between 0.7 and 1.5 Da.

Lipid classes were detected using selective precursor ion scanning (PIS) and constant neutral loss (NL) scanning modes. PIS at *m/z* +184 was used to identify protonated adducts of phosphatidylcholines (PCs), lysophosphatidylcholines (LPCs), and sphingomyelins (SMs), while PIS at *m/z* +264 was applied for protonated ceramides (Cers). Additionally, PIS at *m/z* +369 enabled detection of cholesteryl esters (CEs) as ammonium adducts, and PIS at *m/z* −241 was used to detect deprotonated phosphatidylinositols (PIs). NL scanning identified protonated adducts of phosphatidylethanolamines (PEs) by detecting a loss of 141 Da (NL141), while direct MS+ scanning was used to assay ammonium [M+NH4+] adducts of TAG.

The recorded chromatograms were processed into mzML format using the MSConvert tool from the ProteoWizard toolkit (44). The mzML files were subsequently imported into MZmine2 software (version 2.5.3) (45) for peak picking and identification. Given that the data was acquired by direct infusion MS the full chromatographic range was used for each analysis. Since the data derived from PIS and NL scan acquisition methods, peak detection settings were optimized for clean spectra with wide peak picking windows. Peak alignment was performed based solely on *m/z* values, using the Join aligner module. Compound identification was conducted through a custom database search against curated target databases for each lipid class, which included lipid identification and specific *m/z* values associated with each class. Following peak identification, the intensities of each identified peak were exported as .csv files. The datasets were combined and processed using an Orange3 pipeline (v. 3.34.1, University of Ljubljana, Slovenia) to perform blank corrections, where background contaminants were accounted for based on extraction blanks prepared in 200 mM ammonium formate spiked with the IS cocktail. Lipid quantification then was conducted through a single-point calibration approach, employing one IS per lipid class, which has been shown in previous studies to provide sufficient accuracy for shotgun lipidomics (44). The lipid abundances were calculated from the peak areas relative to the spiked-in ISs, with normalization to the protein content of the original sample to ensure comparability across samples. To improve data accuracy, type 1 ^13^C isotope correction was applied to the identified lipid species using a generic isotopomer model (45). The lipid annotations follow those described previously (46).

### LDL(−) and HDL(−) quantification

Total LDL and HDL from patients and healthy controls were submitted to desalting exchange with buffer A (Tris 10 mM, EDTA 1 mM, pH 7.4) using gel-filtration chromatography with P2 columns (EmpBiotech, Berlin, Germany). LDL and HDL were subfractioned by electric charge using anion-exchange chromatography, as previously described for LDL(−) (47). Briefly, before the chromatography, total LDL and HDL were adjusted to a concentration of 0.2 g/l apoB and 0.2 g/l apoA-I, respectively, in buffer A. The percentage of LDL(−) and HDL(−) was then determined by anion-exchange chromatography in an AKTA-FPLC system (GE Healthcare, Chicago, IL) using a MonoQ™ 5/50 Gl column (GE Healthcare) and a gradient stepwise method. Buffer A was used as binding buffer and buffer B (Tris 10 mM, EDTA 1 mM, NaCl 1 M, pH 7.4) was used to elute the sample from the column. Electropositive fraction was eluted at 0.26 M NaCl, whereas the electronegative fraction was eluted at 0.6 M NaCl. The proportion of LDL(−) and HDL(−) was calculated from the chromatograms by calculating the area (280 nm peaks) versus total LDL and HDL area, respectively. Representative chromatograms are shown in Supplementary Fig. S1A, B.

### LDL susceptibility to oxidation and antioxidant capacity of HDL

LDL and HDL from the patients and controls were desalted against PBS at pH 7.4 using gel-filtration chromatography with a PD10 column (Sephadex G-25, GE Healthcare). LDL oxidation susceptibility and the antioxidant capacity of patients’ HDL against LDL oxidation were evaluated by monitoring conjugated diene formation at 234 nm using a Synergy HT spectrophotometer (BioTek, Winooski, VT). Briefly, LDL was incubated at 60 mg apoB/l with 5 μM of CuSO_4_. HDL was incubated at 60 mg apoA-I/l with and without a standard LDL (obtained from a pool of normolipemic plasma and stored with 10% sucrose at −80°C) at 60 mg apoB/l with 5 μM of CuSO_4_. The LDL susceptibility to oxidation was expressed as the lag phase of the LDL oxidation kinetics (48). The antioxidant capacity of HDL was expressed as the capacity of HDL to prolong the lag phase of standard LDL alone, as previously described (49). The ability to reduce 2,2-diphenyl-1-picrylhydrazyl (DPPH) was also used to assess the antioxidant capacity of HDL against a substrate other than LDL (50). Briefly, HDL at 0.2 g apoA-I/l was incubated with DPPH at 0.2 mM for 30 min at room temperature, and the reduction of DPPH was then evaluated by measuring absorbance at 520 nm using a Synergy HT spectrophotometer (BioTek, Winooski, VT).

### LDL susceptibility to aggregation and HDL capacity to prevent aggregation

The LDL particle size of the LDL aggregates was detected using dynamic light scattering (DLS) in a DLS Wyatt DynaPro Plate Reader II (Wyatt Technology, CA), as previously reported (51). Briefly, LDL at 0.2 g apoB/l was incubated with sphingomyelinase (75 mg/l) at 37°C. Measurements were performed every 30 min for 5 h. Data were collected using Dynamics V7 (Wyatt Technology, CA). Time-aggregated size curves were constructed based on all data points, and the inflection point (midpoint of the sigmoidal curve; EC50) was identified.

LDL(−) was used to evaluate the ability of HDL to prevent LDL aggregation, as it is much more prone to aggregation than native LDL, and HDL can attenuate its aggregation (52). LDL(−) was isolated from total LDL (1.019–1.050 g/ml) in pooled plasma using anion-exchange chromatography in an AKTA-FPLC system (GE Healthcare, Chicago, IL), following the same method described for patients’ LDL(−) isolation, but using a HiLoad 26/10 Q Sepharose column (GE Healthcare) (53) that allows loading a high amount of sample. LDL(−) at 0.2 mg apoB/l was then aggregated by vortexing for 1 min, in the presence or absence of HDL (0.2 mg apoA-I/l) from patients and healthy controls. Lipoprotein aggregation was monitored at 450 nm using a Beckman AD340 spectrophotometer (Beckman Coulter, Brea, CA). The capacity of HDL to prevent aggregation was expressed as inhibition of the increment of absorbance of LDL(−) in the presence of HDL compared to LDL(−) alone.

### Inflammatory effect of LDL and anti-inflammatory capacity of HDL in macrophages

THP1-XBlue-MD2-CD14 cells (THP1-CD14) (Invivogen, San Diego, CA) were used for their enhanced inflammatory response to CD14-TLR ligands, as is LDL(−) (54). The experimental cell conditions are based on those previously used (52, 55). The growth medium for THP1-CD14 monocytes was RPMI 1640 supplemented with 10% FBS and 1% penicillin–streptomycin from Biowest (Nuaillé, France), and with Normocin™ (50 mg/ml), Zeocin™ (100 mg/ml), and G418 (100 mg/ml) antibiotics from Invivogen. THP1-CD14 monocytes were seeded (400,000 cells/well) with growth medium supplemented with phorbol 12-myristate 13-acetate (Sigma-Aldrich, Sant Louis, MO) at 50 μg/l for 24 h to activate their differentiation into macrophages. THP1-CD14 macrophages were incubated for 24 h with: 1) LDL from patients and controls (60 mg apoB/l), 2) LDL(−) isolated from pooled plasma (60 mg apoB/l), 3) HDL from patients and controls (60 mg apoA-I/l), or 4) LDL(−) plus HDL from patients and controls. The incubation medium was RPMI 1640 supplemented with 1% FBS and 1% penicillin–streptomycin. The concentrations of LDL(−) and HDL were chosen according to physiologically plasma proportion of LDL(−) and to those previous studies evaluating the effect of HDL on LDL(−)-induced inflammation (52, 55). After incubation, the cell supernatants were collected, and cytokine release was evaluated using ELISA according to the manufacturer’s instructions: interleukin-1β (Diaclone, Besançon Cedex, France) and sICAM-1 (R&D Systems, Minneapolis, MN). Cell viability was measured with a lactate dehydrogenase kit (Sigma-Aldrich, MO).

### Statistical analysis

Continuous descriptive variables were reported as means and SDs or medians and interquartile ranges if they were not normally distributed. Categorical variables were expressed as counts and percentages. In the tables, differences between the 3 groups were analyzed using ANOVA or the Kruskal–Wallis rank sum test (when a nonparametric test was required). In all Figures and Tables, comparison between two groups was assessed using the Student’s *t* test or the Wilcoxon rank-sum test (when a nonparametric test was required) for continuous variables, and the χ^2^ test was used for categorical variables when the samples were unpaired. A paired *t* test or a Wilcoxon matched-pairs signed-rank test was used when the samples were paired. Correlations between parameters were analyzed using Spearman’s correlation test. Volcano plots and scatter plots using Spearman’s correlation coefficients were performed to analyze the relationships between the function parameters of LDL and specific species in the lipidomic analyses. All Figures, except for lipidomic analyses, are presented in Tukey box-and-whisker plot, where Q1 and Q3 quartiles define the ends of the box, and the whiskers extend to the farthest points that are not outliers (1.5 times the interquartile range). Statistical significance was set at *P* ≤ 0.05 (two-sided) for all the analyses. The analyses were performed using GraphPad Prism 9.0.0 (New York) and RStudio statistical package version 2024.04.1 from R 4.3.2 (a language and environment for statistical computing; R Foundation for Statistical Computing, Vienna, Austria; URL https://www.R-project.org/).

## Results

### Clinical characteristics and lipid profiles

The study population included 37 patients with at least one atherosclerotic plaque causing ≥50% stenosis, 27 patients with carotid stenosis < 50%, and 27 healthy controls. The clinical characteristics of these patients at baseline (7 days after stroke) and the control subjects were as reported previously (20). Table 1 shows the main clinical information recorded at 7 days and 1-year follow-up, including statin treatment before and after the stroke. Notably, all the patients were receiving antiplatelet treatment, and almost all were receiving statins 1 year after stroke.

**Table 1:** Clinical characteristics of the patients

	Stroke Patients at 7 days (n = 64)	Stroke Patients at 1 year (n = 35)	Control Group (n = 27)	*P*
Age, mean (SD)	74.9 (9.7)	73.1 (9.6)	73.8 (5.7)	0.539
Sex (female), n (%)	16 (25)	8 (23)	6 (22.2)	0.949
BMI, mean (SD) (kg/m^2^)	25.56 (3.38)	25.08 (3.40)	27.37 (3.87)	0.077
Regular exercise (PACE ≥4), n (%)	30 (46.9)	18 (51.43)	20 (74.1)	0.056
PREDIMED score, md (IQR)	9 (6–10)	9 (8–10)	8 (7–9)	**0.044**
Current Smoking, n (%)	14 (21.9)	3 (8.57)	0 (0.0)	**0.012**
Hypertension, n (%)	53 (82.8)	15 (42.9)	18 (66.7)	**<0.001**
Diabetes, n (%)	27 (42.2)	7 (20)	5 (18.5)	**0.021**
Dyslipidemia, n (%)	43 (67.19)	4 (11.4)	7 (25.9)	**0.021**
Prior stroke, n (%)	11 (17.2)	-	-	-
Prior antiplatelet therapy, n (%)	37 (57.8)	-	5 (18.5)	**<0.001**
Prior statin therapy, n (%)	35 (54.7)	-	6 (22.2)	**0.005**
Prior antihypertensive therapy, n (%)	45 (76.3)	-	18 (66.7)	0.431
Anticoagulant therapy, n (%)	0 (0)	1 (2.86)	-	0.174
Antiplatelet therapy, n (%)	64 (100)	35 (100)	-	0.437
Statin therapy, n (%)	64 (100)	32 (91.43)	-	**0.017**
Baseline NIHSS, md (IQR)	2 (0–5)	2 (0–4)	-	0.594

IQR, interquartile range; PACE, Physician-based Assessment and Counseling for Exercise; NHISS, National Institutes of Health Stroke Scale. Differences between 3 groups were assessed using one-way ANOVA or Kruskal–Wallis rank-sum test. In the case of 2 groups, Student’s *t* test or the Wilcoxon rank-sum test (when a nonparametric test was required) for continuous variables, and the χ2 test for categorical variables were used to compare the 2 groups; *P* ≤ 0.05 indicates significant differences. Statistical significance is indicated in bold.

Table 2 shows the lipid profile of the study populations. At 7 days, the stroke patients had lower levels of LDL-C and HDL-C, but with a higher LDL-C/HDL-C ratio than the controls. One year after the onset of the stroke, an improved lipid profile was observed, because although TAG and VLDL cholesterol remained similar, patients exhibited higher concentrations of HDL-C, lower concentrations of TC and LDL-C, and lower LDL-C/HDL-C and apoB/apoA-I ratios. ApoJ concentrations increased in the patients’ serum compared with control subjects, but did not diminish at the 1-year follow-up. ApoE concentrations were lower in patients than in controls and remained low 1 year after stroke. No differences in apoC-III levels were observed among the groups. Interleukin-6 levels were significantly higher in patients at 7 days compare to controls, while at 1 year poststroke, the statistical significance was no longer observed.

**Table 2:** Lipid profile, apolipoprotein, and interleukin-6 levels in the study populations

	Stroke Patients at 7 days (n = 64)	Stroke Patients at 1 year (n = 35)	Control Group (n = 27)	*P*
Triacylglycerol (mM), md (IQR)	1.20 (0.96–1.83)	1.08 (0.89–1.23)	1.18 (0.90–1.45)	0.48
Total cholesterol (mM), md (IQR)	3.75 (2.91–4.65) #	3.33 (3.07–4.03) #&	4.64 (4.00–5.88)	**<0.0001**
VLDL-cholesterol (mM), md (IQR)	0.23 (0.18–0.32)	0.22 (0.17–0.25)	0.24 (0.18–0.29)	0.48
LDL-C (mM), md (IQR)	2.41 (1.82–3.29) #	2.01 (1.72–2.55) #&	3.09 (2.58–3.91)	**<0.0001**
HDL-C (mM), md (IQR)	1.03 (0.80–1.32) #	1.18 (0.94–1.47) #&	1.34 (1.15–1.59)	**0.0003**
LDL-C/HDL-C ratio, md (IQR)	2.48 (1.92–3.03) #	1.79 (1.38–2.33) #&	2.25 (1.98–2.80)	**<0.0001**
apoB/apoA-I ratio, md (IQR)	0.57 (0.49–0.66)	0.46 (0.39–0.61) #&	0.52 (0.49–0.67)	**0.0091**
apoC-III (g/l), md (IQR)	0.04 (0.02–0.10)	0.06 (0.04–0.11)	0.07 (0.03–0.11)	0.18
apoE (g/l), m ± SD	0.04 ± 0.02#	0.04 ± 0.02#	0.05 ± 0.02	**0.0111**
apoJ (g/l), md (IQR)	0.18 (0.15–0.21) #	0.18 (0.15–0.20) #	0.14 (0.11–0.17)	**0.0006**
IL6 (ng/l), md (IQR)	0.95 (0.20–2.83) #	0.60 (0.05–2.50)	0.20 (0.05–1.00)	**0.0278**

Differences between 3 groups were assessed using one-way ANOVA or Kruskal–Wallis rank-sum test (*P* is shown in the right column). A paired *t* test or a Wilcoxon matched-pairs signed-rank test (paired data) and a Student’s *t* test or the Wilcoxon rank-sum test (unpaired data) were used to compare 2 groups. Significant differences between 2 groups (*P* ≤ 0.05) are indicated as # versus Control group and 1 year versus 7 days. Statistical significance is indicated in bold.

IQR, interquartile range; md, median.

There were no differences in the parameters shown in Table 2 when patients were divided into 2 subgroups according to stenosis degree ≥50% or ≤50%. No difference was found according to receiving or not pharmacological treatment prior to stroke either (Supplemental Table S3). Similarly, no statistical differences were found for the parameters measured hereafter when considering either the degree of stenosis or prior pharmacological treatment; therefore, all stroke patients were grouped together for analyses.

### Lipid and apolipoprotein composition of lipoprotein fractions

The chemical composition of VLDL is shown in Supplementary Fig. S2. Compared to the control group, the patients’ VLDL showed lower TAG and apoE, and higher apoB content. Since VLDL is a precursor of LDL and also exchanges components with HDL, alterations in VLDL may contribute to changes in the composition of LDL and HDL. No changes were observed in the VLDL composition of patients at 1 year versus 7 days.

**Fig. 1:**
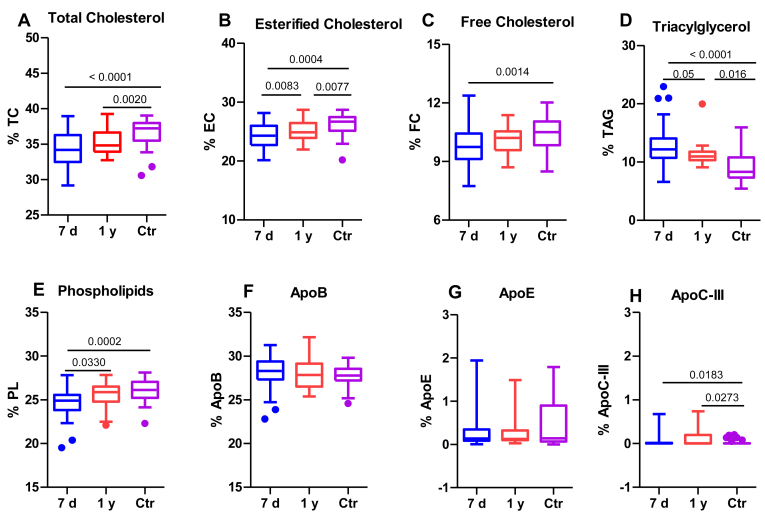
LDL composition. LDL composition was determined using an autoanalyzer, as described in the Methods section. The groups are defined as follows: 7 days represents patients 7 days after ischemic stroke; 1 year represents patients one year after ischemic stroke, and Ctr represents the control group. A: TC, total cholesterol; B: EC, esterified cholesterol; C: FC, free cholesterol; D: TAG, triacylglycerol; E: PL, phospholipid; F: ApoB; and G, ApoE; H: ApoC-III. The results are shown as Tukey box-and-whisker plots. Q1 and Q3 quartiles are the ends of the box, the whiskers extend to 1.5 times de IQR, and dots represent outliers. The results are expressed as percentages of the components in the total lipoprotein mass. A paired *t* test or a Wilcoxon matched-pairs signed-rank test was used when the samples were paired (7 days and 1 year), whereas the Student’s *t* test or the Wilcoxon rank-sum test was used to compare between unpaired samples (7 days versus controls and 1 year versus controls). Horizontal bars indicate statistically significant differences between the groups with *P* ≤ 0.05. IQR, interquartile range.

Figure 1 illustrates several alterations in the composition of patients’ LDL compared to the control group. At 7 days poststroke, patients’ LDL had lower TC (Fig. 1A), both esterified and free forms (Fig. 1B, C), and PL (Fig. 1E) proportion, and higher TAG (Fig. 1D) and apoC-III (Fig. 1H) proportion than in the control group. However, at the 1-year follow-up, the composition of LDL resembled more that observed in the controls, showing higher content in PL and esterified cholesterol, and a decrease in TAG proportion compared to LDL isolated at 7 days, but still showing less TC and esterified cholesterol content than the control group.

**Fig. 2:**
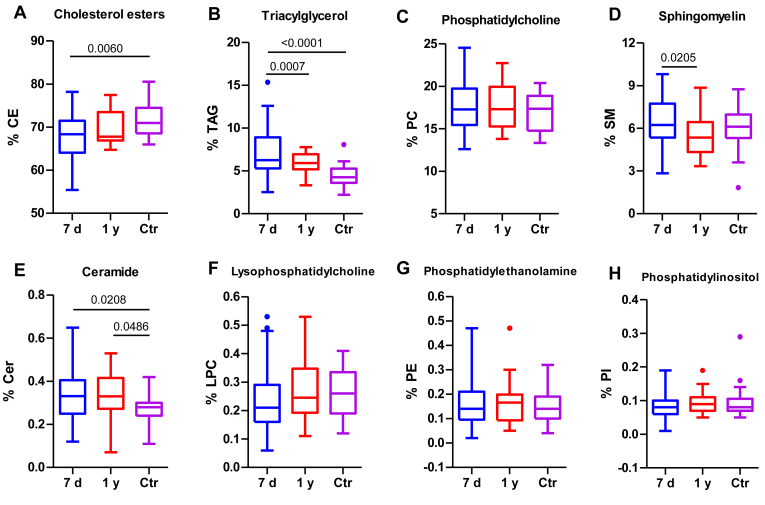
Lipidomic analysis of LDL. Major LDL lipids were determined using lipidomic analysis, as described in the Methods section. The groups are defined as follows: 7 days represents patients 7 days after ischemic stroke; 1 year represents patients 1 year after ischemic stroke, and Ctr represents the control group. A: CE, cholesterol ester; B: TAG, triacylglycerol; C: PC, phosphatidylcholine; D: SM, sphingomyelin; E: Cer, ceramide; F: LPC, lysophosphatidylcholine; G: PE, phosphatidylethanolamine; and H; PI, phosphatidylinositol. The results are shown as Tukey box-and-whisker plots. Q1 and Q3 quartiles are the ends of the box, the whiskers extend to 1.5 times de IQR, and dots represent outliers. The results are expressed as percentages of the components in the total lipoprotein mass. A paired *t* test or a Wilcoxon matched-pairs signed-rank test was used when the samples were paired (7 days and 1 year), whereas the Student’s *t* test or the Wilcoxon rank-sum test was used to compare between unpaired samples (7 days versus controls and 1 year versus controls). Horizontal bars indicate statistically significant differences between the groups with *P* ≤ 0.05. IQR, interquartile range.

To confirm the lipid changes in LDL, and obtain a more detailed knowledge of lipid alterations, a lipidomic analysis was conducted (Fig. 2). A total of 19 species of CE, 24 of TAG, 56 of PC, 29 of SM, 3 of PI, 13 of PE, 2 of LPC, 3 HexosylCer, and 6 of Cer were detected in LDL. The complete list of lipid species and its relative content in each sample can be found as Supplemental Information Data (raw data lipidomics.xls) This analysis confirmed that patients’ LDL had lower CE (Fig. 2A) and higher TAG content (Fig. 2B) than LDL of the control group. One year after stroke, the composition of LDL particles tended to be more similar to that isolated from controls. Regarding the levels of the PL classes, patients’ LDL was enriched in total Cer (Fig. 2E), with this enrichment persisting after 1 year. In contrast, the SM content of patients’ LDL decreased at 1 year poststroke (Fig. 2D).

The changes in the main specific species of CE, triglyceride, and PL species are shown in Supplemental Table S4. This table includes lipid species representing a proportion >0.3% of the total lipids, except for Cers, because of the importance of these species and the existence of significant differences between groups. The levels of the most abundant CE species (CE 18:2) were lower in patients at 7 days than in controls. All triglyceride species were higher in patients at 7 days than in controls. Regarding PC, although several species (PC 36:3, PC 36:4, PC 38:4, and PC 38:6) were higher in patients than in controls, the most abundant specie (PC 34:2) was found decreased. In patients, SM species were similar to controls, but most species diminished 1 year after stroke. Cer 34:1 and Cer 42:2 increases at 7 days compared to controls. These corresponded to Cer 18:1/16:0 and Cer 18:1/24:1, assuming a sphingosine backbone (strongly supported by MS detection in the PIS264 mode). Cer 42:1 (18:1/24:0) remained stable among groups. The Cer 24:1/24:0 ratio was found to be significantly increased in patients at 7 days compared to controls (1.44-fold, *P* = 0.046), but this ratio and the Cer 16:0/Cer 24:0 ratio decreased 1 year after stroke compared to 7 days (*P* = 0.033 and *P* = 0.046, respectively) (Supplemental Fig. S4).

**Fig. 3:**
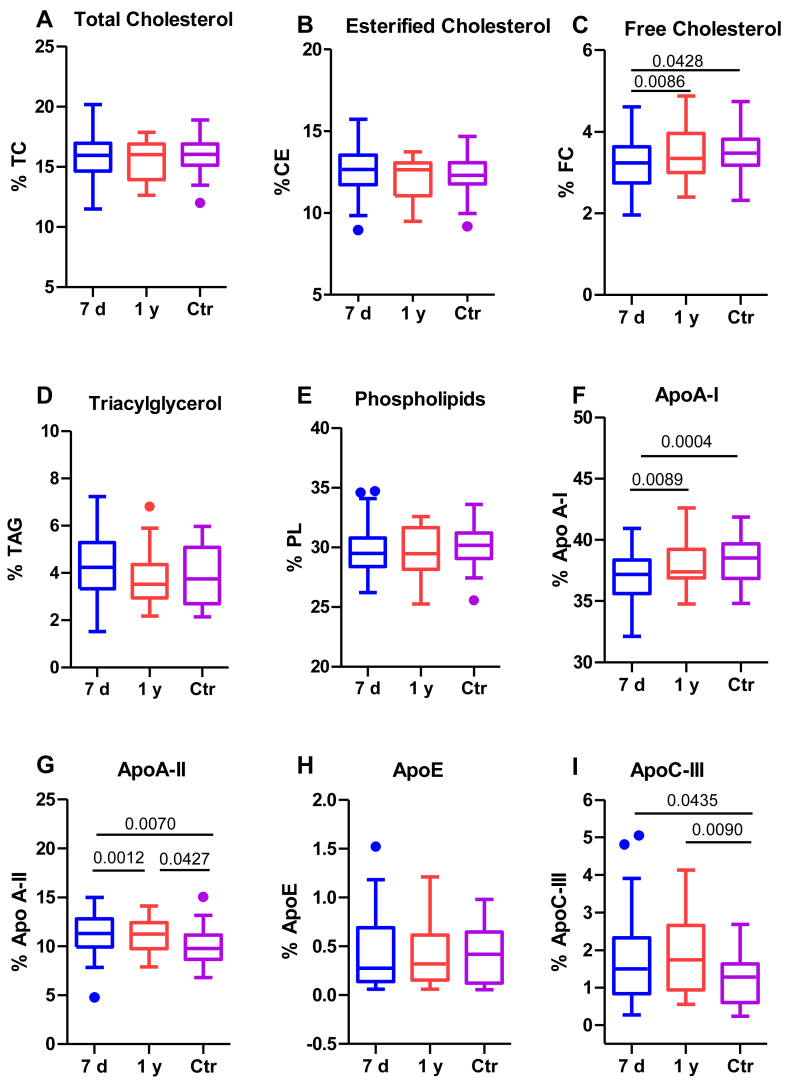
HDL composition. HDL composition was determined using an autoanalyzer, as described in the Methods section. The groups are defined as follows: 7 days represents patients 7 days after ischemic stroke; 1 year represents patients one year after ischemic stroke, and Ctr represents the control group. A: TC, total cholesterol; B: EC, esterified cholesterol; C: FC, free cholesterol; D: TAG, triacylglycerol; E: PL, phospholipids; F: ApoA-I; G: ApoA-II; H: ApoE; and I: ApoC-III. The results are shown as Tukey box-and-whisker plots. Q1 and Q3 quartiles are the ends of the box, the whiskers extend to 1.5 times de IQR, and dots represent outliers. The results are expressed as percentages of the components in the total lipoprotein mass. A paired *t* test or a Wilcoxon matched-pairs signed-rank test was used when the samples were paired (7 days and 1 year), whereas the Student’s *t* test or the Wilcoxon rank-sum test was used to compare between unpaired samples (7 days versus controls and 1 year versus controls). Horizontal bars indicate statistically significant differences between the groups with *P* ≤ 0.05.

Figure 3 shows HDL composition, with patients’ HDL showing no differences in lipid content, except for the lower FC content (Fig. 3C). In contrast, most alterations in patients’s HDL at 7 days were observed in protein cargo, with lower apoA-I content (Fig. 3F) and higher apoA-II (Fig. 3G) and apoC-III content (Fig. 3I) than control subjects. One year after stroke, HDL had higher amounts of FC and apoA-I and lower amounts of apoA-II.

### ApoJ distribution in lipoproteins

**Fig. 4:**
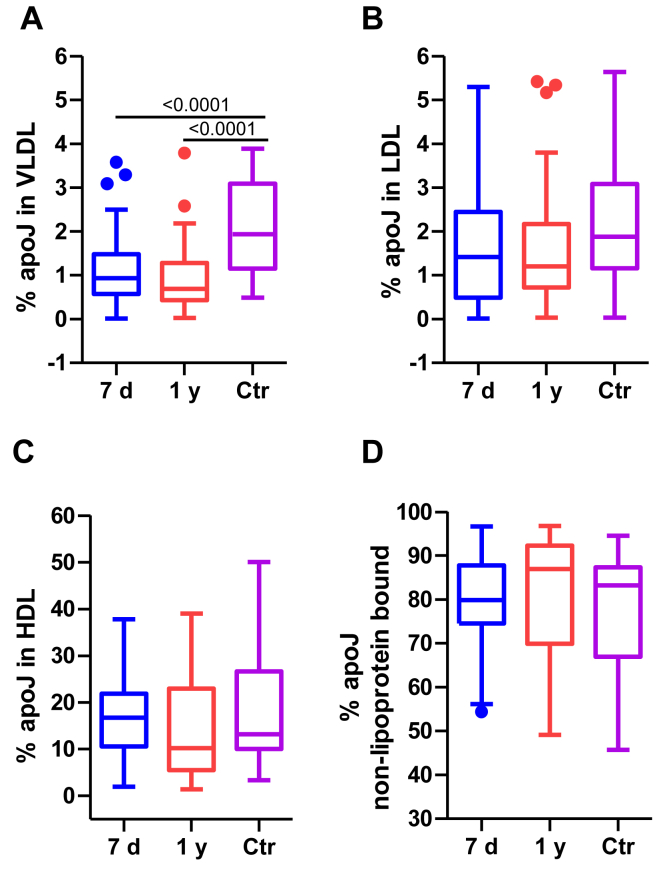
ApoJ distribution in lipoproteins. ApoJ concentration in isolated lipoproteins was quantified using ELISA, as described in the Methods section. The groups are defined as follows: 7 days represents patients 7 days after ischemic stroke; 1 year represents patients 1 year after ischemic stroke, and Ctr represents the control group. The results are expressed as the relative distribution of apoJ concentration in each lipoprotein after normalization by the concentration of cholesterol contained in each lipoprotein (A) VLDL; (B) LDL; (C) HDL; and in nonlipoprotein bound form (D). The results are shown as Tukey box-and-whisker plots. Q1 and Q3 quartiles are the ends of the box, the whiskers extend to 1.5 times de IQR, and dots represent outliers. A paired *t* test or a Wilcoxon matched-pairs signed-rank test was used when the samples were paired (7 days and 1 year), whereas the Student’s *t* test or the Wilcoxon rank-sum test was used to compare between unpaired samples (7 days versus controls and 1 year versus controls). Horizontal bars indicate statistically significant differences between the groups with *P* ≤ 0.05. IQR, interquartile range.

Regarding the distribution of apoJ between the lipoprotein-bound fraction bound and the free form, the only significant difference observed was a lower content of apoJ in VLDL from stroke patients, both 7 days and 1 year after the event, than in controls (Fig. 4A). No statistical differences were found in the percentage of apoJ bound to LDL or HDL (Fig. 4B, C). The fraction of free apoJ showed a slight, nonsignificant trend toward being elevated in patients at 7 days compared to controls (Fig. 4D).

### Electric charge and size

**Fig. 5:**
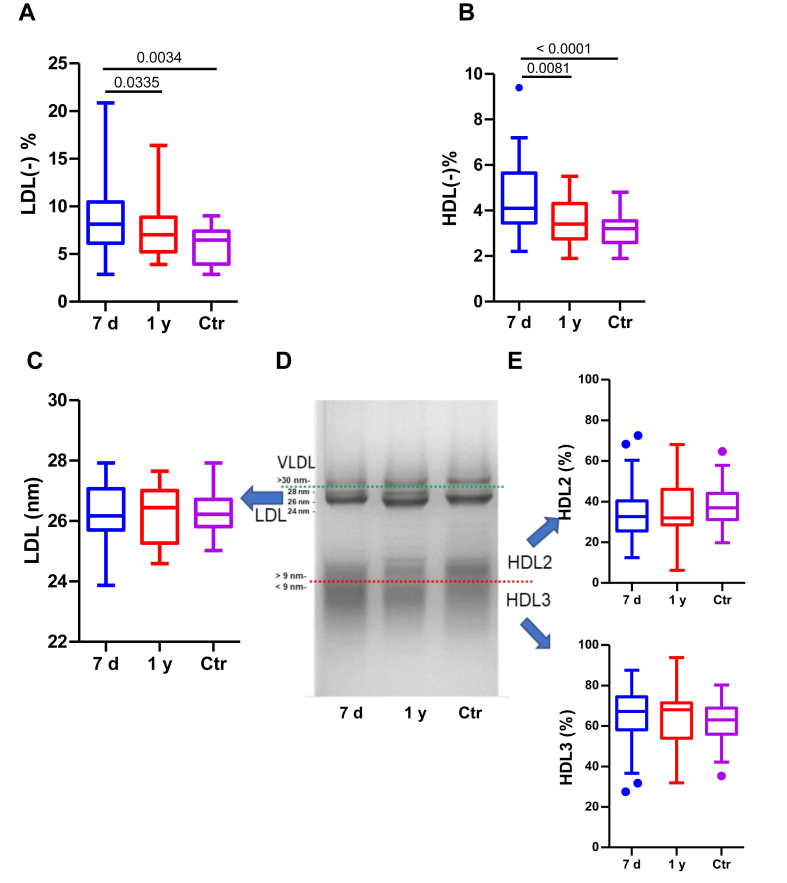
Electronegative charge and particle size of LDL and HDL. The proportion of electronegative lipoproteins: LDL(−) (A) and HDL(−) (B) was determined using anion-exchange chromatography. LDL particle size (C) and the proportion of HDL2 and HDL3 (E) was evaluated using GGE (D). A representative GGE showing LDL and HDL size is shown (D). The groups are defined as follows: 7 days represents patients 7 days after ischemic stroke; 1 year represents patients 1 year after ischemic stroke, and Ctr represents the control group. The results are shown as Tukey box-and-whisker plots. Q1 and Q3 quartiles are the ends of the box, the whiskers extend to 1.5 times de IQR, and dots represent outliers. A paired *t* test or a Wilcoxon matched-pairs signed-rank test was used when the samples were paired (7 days and 1 year), whereas the Student’s *t* test or the Wilcoxon rank-sum test was used to compare between unpaired samples (7 days versus controls and 1 year versus controls). Horizontal bars indicate statistically significant differences between the groups with *P* ≤ 0.05. GGE, gel electrophoresis.

Changes in the chemical composition of LDL and HDL can lead to alterations in their electric charge and particle size. Previously, we demonstrated that our cohort of patients had a higher proportion of LDL(−) (20), as indicated in Figure 5A. Figure 5B shows that patients also had higher proportions of HDL(−) than the controls. Interestingly, significant decreases in both electronegative lipoproteins were observed 1 year after the stroke event, (Fig. 5A, B). HDL(−) proportions at 7 days correlated negatively with apoA-I concentrations in serum (*r* = −0.385, *P* = 0.0011) and almost correlated significantly with LDL(−) concentrations (*r* = 0.233, *P* = 0.055).

Although the patients’ lipoproteins exhibited several differences in their chemical composition and negative charge, LDL size (Fig. 5C) and HDL2/HDL3 proportion (Fig. 5E), both evaluated using GGE, were not altered in patients versus controls and remained stable at 1 year after stroke. A representative GGE is shown in Figure 5D.

### Susceptibility to oxidation and aggregation of LDL

**Fig. 6:**
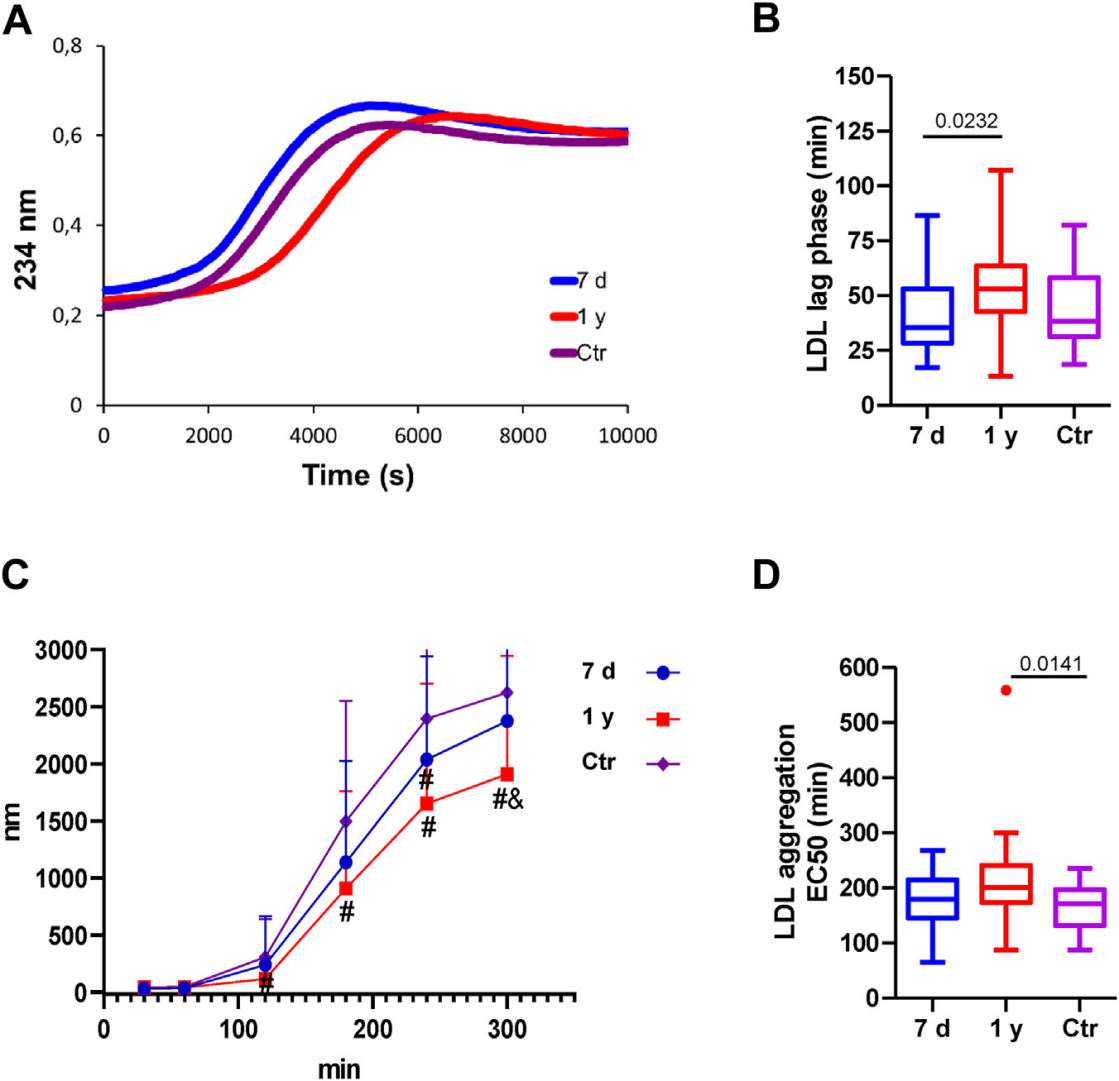
Susceptibility to oxidation and aggregation of LDL. The susceptibility of LDL to oxidize was evaluated by diene formation kinetics after copper-induced oxidation and the susceptibility to aggregate by kinetic measurement of the particle size by DLS after SMase-induced aggregation as described in the Methods section. The parameters represented are (A) Representative LDL diene formation kinetics; (B) LDL lag phase of the diene formation kinetics; (C) kinetics showing the size of LDL quantified by DLS after the induction of aggregation; and (D) EC50 of the LDL aggregation kinetics using DLS. The groups are defined as follows: 7 days represents patients 7 days after ischemic stroke; 1 year represents patients one year after ischemic stroke, and Ctr represents the control group. The results are shown as Tukey box-and-whisker plots. Q1 and Q3 quartiles are the ends of the box, the whiskers extend to 1.5 times de IQR, and dots represent outliers. A paired *t* test or a Wilcoxon matched-pairs signed-rank test was used when the samples were paired (7 days and 1 year), whereas the Student’s *t* test or the Wilcoxon rank-sum test was used to compare between unpaired samples (7 days versus controls and 1 year versus controls). Horizontal bars indicate statistically significant differences between the groups, in C) # versus Control group and 1 year versus 7 days, *P* ≤ 0.05. DLS, dynamic light scattering; IQR, interquartile range.

We previously observed increased concentration of serum oxidized LDL and decreased activity of enzymes related to the prevention of lipoprotein oxidation in the non-apoB fraction of serum from patients (20). However, in the present study, we found that LDL from patients was not more prone to *in vitro*–induced oxidation than LDL from controls, even becoming more resistant to oxidation 1 year after stroke (Fig. 6A, B). Regarding LDL’s aggregability, no difference was observed between patients at 7 days and the control group (Fig. 6C, D). Notably, patients’ LDL became more resistant to aggregation 1 year after stroke.

### Protective actions of HDL against oxidation and aggregation

**Fig. 7:**
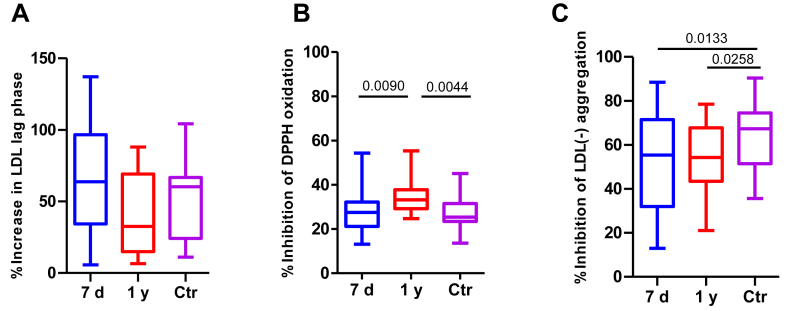
Protective actions of HDL against oxidation and aggregation. The protective actions of HDL against the modification of LDL were evaluated as described in the Methods section. The figures represent the following effects promoted by HDL (A) increase in the lag phase of diene formation kinetics; (B) inhibition of DPPH oxidation, quantified by colorimetric detection after 30 min of incubation; and (C) inhibition of LDL(−) aggregation induced by vortexing. The results are expressed as % of increase (A) or % of inhibition (B and C) in the presence versus the absence of HDL. The groups are defined as follows: 7 days represents patients 7 days after ischemic stroke; 1 year represents patients one year after ischemic stroke, and Ctr represents the control group. The results are shown as Tukey box-and-whisker plots. Q1 and Q3 quartiles are the ends of the box, the whiskers extend to 1.5 times de IQR, and dots represent outliers. A paired *t* test or a Wilcoxon matched-pairs signed-rank test was used when the samples were paired (7 days and 1 year), whereas the Student’s *t* test or the Wilcoxon rank-sum test was used to compare between unpaired samples (7 days versus controls and 1 year versus controls). Horizontal bars indicate statistically significant differences between the groups with *P* ≤ 0.05. DPPH, 2,2-diphenyl-1-picrylhydrazyl; IQR, interquartile range.

The parameters related to the antioxidant properties of HDL and against in vitro LDL aggregation are shown in Figure 7. The capacity of HDL to lengthen the kinetic of diene formation in LDL did not differ among the groups (Fig. 7A). On the other hand, at 1 year after stroke, HDL from patients reduced DPPH more efficiently than at 7 days and even more efficiently than HDL in the control group (Fig. 7B). Regarding aggregation, patients’ HDL showed a weaker ability to counteract the aggregation of LDL(−) than controls’ HDL, both at 7 days and 1 year after stroke (Fig. 7C).

### Inflammation in macrophages

**Fig. 8:**
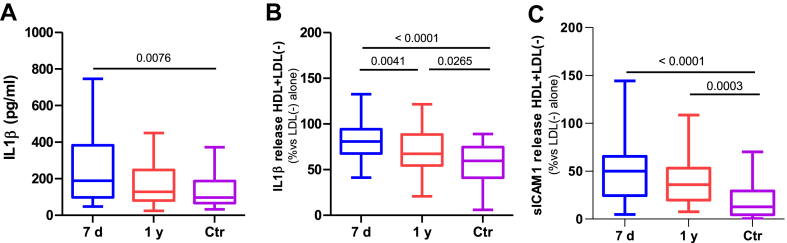
Proinflammatory and anti-inflammatory effects of LDL and HDL on macrophages. Macrophages were incubated with lipoproteins and molecules in the supernatant were quantified using ELISA. The inflammatory capacity of LDL was determined by the release of IL1β (Α). The capacity of HDL to prevent LDL(−)-induced effects was expressed in percentage as the release of IL1β (B) and sICAM-1 (C) in the presence of HDL versus the release promoted by LDL(−) alone. The groups are defined as follows: 7 days represents patients 7 days after ischemic stroke; 1 year represents patients one year after ischemic stroke, and Ctr represents the control group. The results are shown as Tukey box-and-whisker plots. Q1 and Q3 quartiles are the ends of the box, the whiskers extend to 1.5 times de IQR, and dots represent outliers. A paired *t* test or a Wilcoxon matched-pairs signed-rank test was used when the samples were paired (7 days and 1 year), whereas the Student’s *t* test or the Wilcoxon rank-sum test was used to compare between unpaired samples (7 days versus controls and 1 year versus controls). Horizontal bars indicate statistically significant differences between the groups with *P* ≤ 0.05. IQR, interquartile range.

Patients’ LDL induced greater IL1β release from macrophages than LDL from controls (Fig. 8A). This inflammatory effect was observed without changes in cell viability, which remained higher than 80% across the groups. LDL-induced IL1β release correlated with the content in LDL of Cer 42:2 (r = 0.264, *P* = 0.02), and Cer 42:1 (r = 0.242, *P* = 0.04), and was inversely correlated with the lag phase of LDL oxidation (r = −0.50, *P* = 0.0012).

The anti-inflammatory effect of HDL, which prevents the LDL(−)-induced cytokine release in macrophages, was compromised in the patients. The inhibitory effect of HDL on the LDL(−)-induced release of IL1β (Fig. 8B) and sICAM-1 (Fig. 8C) was lower in HDL from patients at 7 days than in HDL from control subjects. However, patients’ HDL recovered some of its anti-inflammatory effect 1 year after stroke, with a significant improvement in inhibiting IL1β release compared to 7-day samples, albeit less potently than control HDL.

### Association between LDL lipidome and LDL function

**Fig. 9:**
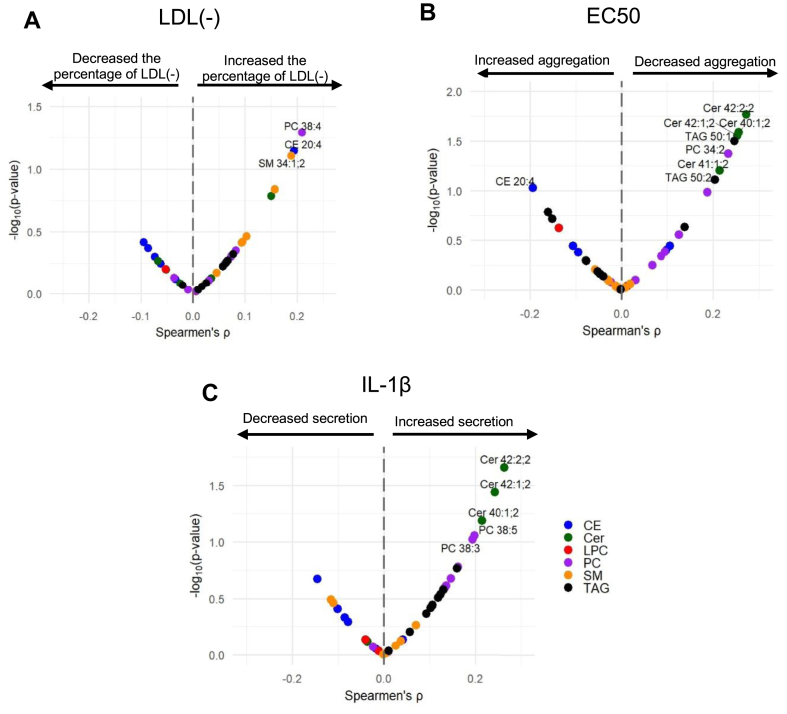
Relationship between the LDL functional parameters and lipid species abundances by volcano plot. Volcano plots showing the relationship (Spearman correlation coefficients) between the function parameters of LDL (electronegativity (A), aggregation (B), and inflammation (C)) and LDL lipidome were done as described in the Methods section. Lipid species with −log10 *P* values >1.0 (*P* value ≤ 0.1) have been written in full. CE, cholesteryl ester; Cer, ceramide; LPC, lysophosphatidylcholine; PC, phosphatidylcholine; SM, sphingomyelin; TAG, triacylglycerol.

To further analyze the relationship between LDL lipidome and LDL function, we conducted correlation analyses. Volcano plots were used to identify associations between specific lipid species and the functional parameters of LDL that were altered in the patients (Fig. 9). No association was observed between lipid species content and lag phase time regarding susceptibility to oxidation (not shown). PC 38:4, CE 20:4, and SM 34:1 content correlated positively with LDL(−) (*P* ≤ 0.05) (Fig. 9A). Several lipid species, most notably certain Cer and TAG species, were associated with decreased LDL aggregation (Fig. 9B), whereas only CE 20:4 appeared to be positively associated. Interestingly, the Cer species associated with low LDL aggregation were also associated with increased proinflammatory capacity (Fig. 9C).

**Fig. 10:**
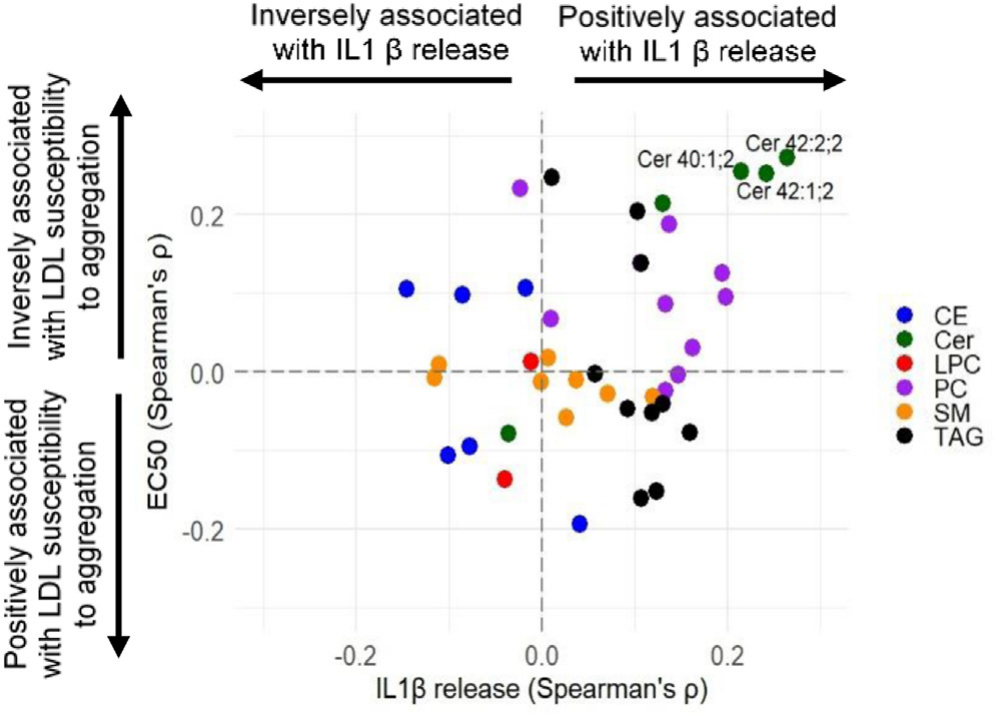
Relationship between the LDL functional parameters and lipid species abundances by scatter plots. The scatter plot shows the correlation coefficients for the Spearman’s rank correlation analysis of LDL lipid species abundances versus LDL’s potency to elicit macrophage IL1β release (x) and of LDL lipid species abundance versus LDL aggregation (EC50) (y). Lipid species with *P* > or < ± 0.2 have been written in full. We have written out the number of times ± 0.2. CE, cholesteryl ester; Cer, ceramide; LPC, lysophosphatidylcholine; PC, phosphatidylcholine; SM, sphingomyelin; TAG, triacylglycerol.

To confirm the dual role of Cer species (Cer 40:1, Cer 42:1, and Cer 42:2) in LDL aggregation and inflammatory capacity, we conducted multivariate Spearman’s correlation analyses. We computed correlation coefficients between the abundances of LDL lipids and two key functional parameters: the aggregation propensity of LDL and its potency to elicit IL1β release from macrophages. The results are presented as bivariate correlation scatter plots in Figure 10. This plot illustrates the relationship between LDL lipid abundance and their functional effects. It can be observed that the three Cer species correlated positively with the capacity of LDL to induce IL1β release. Conversely, these Cer species correlated negatively with the parameters that indicated susceptibility to aggregation.

## Discussion

Although the role of lipoproteins in atherosclerosis is well known, their specific involvement in ischemic stroke associated with atherosclerosis has not been fully elucidated. The present study encompassed extensive compositional and functional analyses of the lipoprotein fractions and revealed alterations in lipoproteins from ischemic stroke patients after the vascular event. Our findings demonstrate that 1) LDL from patients 7 days poststroke had increased electronegative charge, altered composition, and induced inflammation in macrophages; 2) HDL from patients 7 days after stroke had an increased electronegative charge and exhibited several changes in the apolipoprotein cargo, resulting in a diminished protective effect against LDL modification and LDL(−)-induced inflammation; and 3) One year poststroke, the LDL and HDL compositions shifted closer to those of the controls. LDL became less prone to modification, and HDL regained its protective antiatherosclerotic properties.

Our cohort of patients displayed normal lipid profile after the stroke, with lower TC and LDL-C levels than the healthy controls. This observation aligns with the known lipid-lowering effect attributable to the stroke event (56). However, the lipid and apolipoprotein composition in the lipoproteins of these patients was greatly altered. On the other hand, our findings contradict previous studies reporting changes in LDL and HDL size, such as the presence of large-sized HDL in stroke patients (30, 31, 35) and increased levels of small-dense LDL particles associated with an increased risk of ischemic stroke (57), owing to different study populations and experimental conditions. Despite the normal size of LDL and HDL in our patients, there was an increase in their electronegative charge, probably due to the observed altered biochemical composition and/or potential structural changes. Since composition, charge, and function of lipoproteins are tightly intertwined, the altered composition and increased negative charge of LDL and HDL in patients seemed to drive the generation of dysfunctional lipoproteins.

Patients’ VLDL exhibited lower apoE content than the control group. This reduction could have blunted VLDL clearance, which may have contributed to the presence of altered LDL and HDL. Such changes collectively lead to increased CV risk (58, 59). VLDL also showed lower apoJ content in patients than controls. In contrast, patients had higher plasma concentrations of total apoJ than controls. This altered apoJ distribution, with higher total apoJ concentration in serum but lower lipoprotein-associated content, has been previously described in patients with conditions associated with CV risk, such as dyslipidemia or obesity (19, 60).

Notable lipid alterations were observed in patients’ LDL, which showed lower TC and PL and higher TAG and Cer contents. In Nieddu *et al.*’s study, LDL in patients undergoing carotid endarterectomy had lower content of certain lipid species (PE 38:6, SM 32:1, SM 32.2) associated with the presence of soft hypoechoic carotid plaques (34). The present study revealed no differences in these specific lipid species, probably owing to the 2 studies having different type of populations. In contrast, LDL from our patients was enriched in Cer species (Cer 16:0 and Cer 24:1) related to CV risk (61, 62). The ratio between proatherogenic and antiatherogenic Cer was also increased in LDL from patients, but it decreased after 1 year. We found that increased Cer 24:1 content in LDL correlated with the induction of IL1β release in macrophages, which agreed with previous reports that Cer content determines the inflammatory potential of LDL (63, 64).

The increased Cer content in LDL isolated from stroke patients at 7 days seemed to contradict the lack of differences in the aggregability of LDL of these subjects compared to controls. However, other alterations in patients’ LDL, such as having higher TAG content than controls, could explain this discrepancy, because such molecule confer protection toward LDL aggregation (65). The increased apoC-III content may also have influenced LDL properties, as apoC-III binds preferentially to TAG-enriched LDL particles, leading to the inflammatory action of LDL and to increased CV risk (66). Thus, TAG-enriched LDL levels have been robustly associated with an increased risk of ischemic stroke (67).

In this study, we have to consider that, at the stroke onset, the patients received high doses of statins, whereas only 54.7% were under statin therapy before the event. Beyond lipid profile, statins have multiple beneficial effects, such as anti-inflammatory potential and the ability to counteract lipoprotein modification (68). Some of their protective actions, such as anti-inflammatory response and reduction of LDL(−), can occur as quickly as 5 days (69, 70). Although in our study plasma levels of the inflammatory interleukin-6 at 7 days were higher than in controls, these values may have been even higher during the acute phase. Statins could also improve some lipoprotein-related features, minimizing the alterations or differences compared to healthy controls at 7 days. The influence of the pharmacological treatment is likely stronger at 1 year, as 91.43% of the patients adhered to statin therapy and all were under antiplatelet treatment. In this regard, at 3 and 6 months of therapy, simvastatin progressively decreases the proportion of LDL(−) and positively modifies LDL lipid content (71). Antiplatelet agents have also been shown to exert significant beneficial effects on plasma lipids (72). Additionally, improvements in healthy habits, such as smoking cessation at 1 year, likely contributed to better lipoprotein features. Altogether, these factors may have partly accounted for the lack of differences between patients and controls in LDL aggregation and oxidation, as well as lipoproteins properties, particularly after 1 year of follow-up.

Some of the alterations in the lipid and apolipoprotein content of patients’ LDL (TAG, apoC-III, Cer) concurred with the chemical composition ascribed to LDL(−) (21, 63, 73, 74). Accordingly, this cohort of patients showed an increased proportion of LDL(−), which decreased 1 year after stroke in parallel with a partial restoration of the compositional features of LDL. Since LDL(−) is an inflammatory inductor (21), it may have contributed to the IL1β release promoted by LDL from patients at 7 days. However, no correlation was found between IL1β release and LDL(−), whereas there was a correlation with lag phase of LDL oxidation, suggesting that susceptibility to oxidation of LDL was related to the induction of inflammation in macrophages.

Regarding HDL, the lipid composition was scarcely modified in patients, except for FC diminution, but it had an altered content in several apolipoproteins, particularly higher apoC-III, and apoA-II, and lower apoA-I content. Several studies have shown that changes in HDL proteome are associated with dysfunctional HDL and CVD (75, 76). Consequently, some studies have reported a loss of protective action by HDL in stroke patients (30, 31, 35, 77). The impaired HDL properties have been related to proteomic alterations, such as high apoJ (35) and serum amyloid A (33) content or decreased apoA-I content (30). ApoC-III enrichment in HDL is another alteration that has been related to impaired functionality (78). Those studies and the abnormalities found in the apolipoprotein content of HDL in our cohort of patients suggested that its protective action had been altered.

Moreover, patients’ HDL had a higher proportion of HDL(−). Previously, other authors have reported the presence of HDL with increased negative charge in diseases related to inflammation (79, 80). These particles showed impaired protective actions regarding cholesterol efflux, anti-inflammatory, and anti-apoptotic actions, even promoting inflammation and foam cell formation (80). HDL’s electronegativity was partially attributed to a higher amount of apoC-III, as the percentage of apoCIII-containing HDL particles correlated with a lack of atheroprotective action and coronary artery disease risk (78, 81). Notably, in our study, the increased proportion of HDL(−) in stroke patients was not related to the amount of circulating HDL.

Patients’ HDL showed impaired protective action against the in vitro effect of LDL(−), especially in the inhibition of its high susceptibility to aggregation and the induction of cytokine release in macrophages. This loss of protective action may have resulted from the lower apoA-I/apoA-II ratio and the higher apoC-III content. These or other chemical changes could have altered the structure of HDL affecting its ability to bind to LDL(−) and/or to bind bioactive molecules, such as Cer or free fatty acids, from LDL(−). Regarding the antioxidant ability of patients’ HDL, no significant alterations were found in avoiding conjugated diene formation in LDL, in agreement with a previous study (31). The antioxidant ability of HDL against DPPH remained unaltered at 7 years compared to control subjects either.

Interestingly, 1 year after the stroke, HDL from patients gained antioxidant capacity against DPPH and anti-inflammatory potential against the LDL(−)-induced effect on macrophages. It has been reported that, in the acute phase of ischemic stroke, the existing inflammatory milieu promotes remodeling of the HDL proteome leading to HDL dysfunctionality (35). Accordingly, our results 1 year after stroke suggest that when the acute inflammatory state is overcome the composition of patients’ HDL restores, improving its function. In our case, the changes in apoC-III and the apoA-I/apoA-II ratio may have contributed to the partial recovery of HDL function. Patients’ shift to healthy habits and their intensive medical treatment, including statin therapy, may have contributed to the improvement in the lipoproteins’ function.

Our study has some limitations that compel consideration, particularly the relatively small number of patients and the incomplete samples collection during the follow-up. At 1 year, there is an unavoidable bias, owing to carotid endarterectomy, death, stroke recurrence or other CV events, which exclude patients who did not have a favorable follow-up. These factors limit the generalizability of the findings to larger populations and the restrict subgroup analyses by sex or specific pharmacological treatments. Although some of the differences found in LDL from patients are in minor lipids, such as Cer, modifications in the content of minor components can elicit great alterations in LDL function. Another limitation is the lack of lipidomic studies in HDL, which were not performed due to the absence of differences in lipid content as assessed by the autoanalyzer. A factor likely influencing lipoprotein behavior, as discussed earlier, is statin treatment; however, the use of this therapy after stroke is mandatory according to clinical guidelines. Other aspects, such as the molecular mechanisms activated by altered lipoproteins and whether their compromised qualitative properties are a result of the stroke or preexisting, remain uncertain and will be addressed in future studies.

The importance of the present study lies in the influence of the qualitative properties of lipoproteins in CVD and the fact that these alterations can be reverted. Studies by Ruuth *et al.* have shown that pharmacological treatment and healthy diet can favorably modify the LDL lipidome, reducing LDL’s susceptibility to modification and potentially lowering CV risk (65, 82, 83). In the search for clinically relevant biomarkers, LDL(−) has been proposed as a biomarker for predicting CVD, including in asymptomatic patients (84, 85). The effects and potential role of HDL(−) as a biomarker have been less studied, but it remains a promising topic. According to our results, the determination of these molecules and/or other qualitative lipoprotein properties, alongside inflammatory biomarkers, could be useful in predicting the risk of first-ever stroke or recurrence, and/or in evaluating treatment efficacy. While routine determination of these parameters may be less practical than automated blood measurements, they could serve as valuable alternative or additional markers in certain patients at residual CV risk who might benefit from tailored interventions. Furthermore, studying the molecular and cellular mechanisms activated by altered lipoproteins in stroke (such as more electronegative or altered composition) could pave the way for strategies aimed at improving their properties and mitigating the resulting harmful effects.

In summary, these results emphasize the qualitative alterations found in LDL and HDL from ischemic stroke patients with carotid atherosclerosis. The altered lipoproteins’ function hinged on changes in their biochemical composition and electric charge. HDL in patients lost some of its protective properties, including the capacity to prevent LDL aggregation and LDL-induced inflammation. Notably, several of the alterations observed in lipoproteins were reversed 1 year after the stroke onset in those patients with a favorable follow-up. Although the results obtained are not expected to have immediate clinical applicability, the determination of some of these parameters may prove useful in the future for monitoring disease activity or response to treatments. Further studies in larger cohorts are warranted to gain deeper insights into the lipoprotein-related modifications underlying ischemic stroke, including the role of LDL(−) and HDL(−) in atherosclerotic-related processes.

## Data availability

All the information of this study is available upon reasonable request by contacting with the corresponding author.

## Supplemental data

This article contains supplemental data.

## Conflict of interest

S. B., N. R., and J. L. S.-Q. are members of the CIBER of Diabetes and Metabolic Diseases (CIBERDEM; CB07/08/0016). S. B. and N. R., are members of EpiLipidNET COST action, and S. B., P. C.-R., J. M.-F., and A. A.-S. are members of RICORS-ICTUS (RD21/0006/0006). S. B., J. L. S.-Q., N. P., and N. R. are members of the Quality Research Group 2017-SGR-1149 from 10.13039/501100002809Generalitat de Catalunya, and they are members of the Spanish Atherosclerosis Society Vascular Biology Group. The other authors declare that they have no conflicts of interest with the contents of this article.

## Supplementary data

Supplemental material

Raw data Lipidomics
