# Supplementary material for: Alterations in LDL and HDL after an ischemic stroke associated with carotid atherosclerosis are reversed after 1 year
Source: J Lipid Res. 2024 Dec 31;66(2):100739. doi: 10.1016/j.jlr.2024.100739 (PMC11815653; doi:10.1016/j.jlr.2024.100739)
Supplement: Supplemental material [file mmc1.docx]

**Supplemental Tables**

Supplemental Table S1. Internal lipid standards and their spiked amounts

| **Lipid class** | **Lipid species** | **mass (Da)** | **pmol spike** |
| --- | --- | --- | --- |
| CE | CE 18:1(+[2]H7) | 657,644 | 5830 |
| PC | PC 15:0/18:1(+2[H]7) | 752,606 | 2126 |
| PE | PE 15:0/18:1(+2[H]7) | 710,559 | 70 |
| PS | PS 15:0/18:1(+2[H]7) | 754,549 | 66 |
| PI | PI 15:0/18:1(+2[H]7) | 829,572 | 120 |
| SM | SM 18:1;2/18:1(+2[H]9) | 739,655 | 408 |
| DAG | DAG 15:0/18:1(+2[H]7) | 587,551 | 10 |
| TAG | TAG 15:0/18:1(+2[H]7)/15:0 | 811,765 | 700 |
| LPC | LPC 18:1(+[2]H7) | 528,392 | 406 |
| Cer | Cer 18:1;2/12:0 | 488,493 | 500 |

|  |  |  |  |  |  |  |  |  |  |  |  |  |  |  |
| --- | --- | --- | --- | --- | --- | --- | --- | --- | --- | --- | --- | --- | --- | --- |
| **Lipid class** | **Acquisition**  **mode** | **Polarity** | **Capillary (V)** | **Gas**  **temperature (℃)** | **Gas flow**  **(L/min)** | **Nebulizer**  **(psi)** | **Range low**  **(m/z)** | **Range high**  **(m/z)** | **Step size**  **(amu)** | **Scan time**  **(ms)** | **Collision energy**  **(V)** | **Fragmentor (V)** | **Cell accelator (V)** | **Multiplier offset** |
| TAG | MS+ | pos | 4000 | 250 | 11 | 20 | 400 | 1000 | 0,1 | 2000 | - | 380 | 4 | 400 |
| CE | P369 | pos | 5000 | 250 | 3 | 40 | 300 | 950 | 0,1 | 2800 | 13 | 135 | 5 | 400 |
| PC, LPC, SM | P184 | pos | 5000 | 250 | 3 | 40 | 300 | 950 | 0,1 | 2800 | 30 | 250 | 5 | 400 |
| Cer | P264 | pos | 5000 | 250 | 3 | 40 | 400 | 950 | 0,1 | 2800 | 30 | 75 | 5 | 400 |
| PE | NL 141 | pos | 5000 | 250 | 3 | 40 | 300 | 900 | 0,1 | 2500 | 25 | 135 | 5 | 400 |
| PI | P241 | neg | 5000 | 250 | 3 | 40 | 650 | 1100 | 0,1 | 3000 | 48 | 360 | 5 | 400 |

Supplemental Table S2. Instrument paràmetres for shotgun lipidomics

*Instrument***:** Agilent 6410 Triple quadrupole (Agilent Technologies)

*Acquisition software:* Agilent MassHunter Workstation (version B.01.03)

*Source:* ESI

Suplemmental Table S3. Lipid profile,apolipoprotein and IL-6 levels in the stroke patients at 7 days according to prior statin therapy.

|  | Stroke patients with prior statin therapy (n=35) | Stroke patients without prior statin therapy (n=29) | p |
| --- | --- | --- | --- |
| Triacylglycerol (mM), md (IQR) | 1.91 (0.92-1.59) | 1.10 (0.96-1.63) | 0.622 |
| Total cholesterol (mM), md (IQR) | 3.62 (2.83-4.19) | 4.38 (3.05-4.90) | 0.051 |
| VLDL-C (mM), md (IQR) | 0.24 (0.18-0.32) | 0.22 (0.19-0.33) | 0.622 |
| LDL-C (mM), md (IQR) | 2.31 (1.74-2.74) | 3.02 (1.91-3.48) | 0.055 |
| HDL-C (mM), md (IQR) | 1.01 (0.76-1.33) | 1.03 (0.81-1.28) | 0.891 |
| LDL-C/HDL-C ratio, md (IQR) | 2.42 (1.85-3.93) | 2.64 (2.01-3.27) | 0.182 |
| apoB/apoA-I ratio, m ± sd | 0.574 ± 0.125 | 0.583 ± 0.178 | 0.820 |
| apoC-III (g/l), md (IQR) | 0.06 (0.03-0.08) | 0.03 (0.02-0.11) | 0.745 |
| apoE (g/l), md (IQR) | 0.04 (0.03-0.06) | 0.03 (0.02-0.04) | 0.400 |
| apoJ (g/l), md (IQR) | 0.17 (0.14-0.22) | 0.19 (0.15-0.22) | 0.418 |
| IL6 (ng/l), md (IQR) | 0.80 (0.18-1.53) # | 1.60 (0.28-6.25) | 0.092 |

*Differences between groups were assessed using Wilcoxon rank-sum test or Wilcoxon test. p ≤ 0.05 indicates significant differences between groups.*

Supplemental Table S4. Relative proportion of the main CE, TG, and PL species versus total lipids in LDL

|  | Stroke patients  7 days (n=64) | Stroke patients  1 year (n=35) | Control group (n=27) | p |
| --- | --- | --- | --- | --- |
| CE 16:0 | 5.640 (5.055-6.700) | 5.923 ± 1.794 | 5.458 ± 1.967 | 0.595 |
| CE 18:1 | 12.570 ± 1.737 | 13.550 ± 2.731 | 13.240 ± 2.184 | 0.168 |
| CE 18:2 | 32.660 ± 5.104 # | 34.460 ± 5.288 # | 39.360 ± 4.653 | 0.0002 |
| CE 18:3 | 1.13 (0.925-1.260) | 1.405 ± 0.382 & | 1.180 (0.990-1.605) | 0.0233 |
| CE 20:4 | 9.360 ± 1.850 # | 8.650 ± 2.058 # | 7.467 ± 1.810 | 0.001 |
| CE 20:5 | 0.850 (0.655-1.018) | 0.750 (0.540-1.223) | 0.910 (0.590-1.255) | 0.948 |
| TG 52:2 | 1.390 (0.965-1.870) # | 0.945 (0.862-1.475) # | 0.889 ± 0.322 | 0.0009 |
| TG 52:3 | 1.080 (0.840-1.430) # | 0.861 ± 0.226 # | 0.630 (0.585-0.785) | < 0.0001 |
| TG 52:4 | 0.410 (0.305-0.490) # | 0.329 ± 0.128 # | 0.210 (0.175-0.255) | < 0.0001 |
| TG 54:3 | 0.664 ± 0.291 # | 0.565 ± 0.206 # | 0.378 ± 0.124 | 0.0001 |
| TG 54:4 | 0.540 ± 0.215 # | 0.432 ± 0.122 # | 0.260 (0.230-0.345) | < 0.0001 |
| TG 54:5 | 0.310 (0.225-0.385) # | 0.271 ± 0.090 # | 0.170 (0.135-0.200) | < 0.0001 |
| PC 34:1 | 2.180 (1.835-2.925) | 2.334 ± 0.673 | 2.317 ± 0.510 | 0.960 |
| PC 34:2 | 3.300 (2.690-3.870) # | 3.264 ± 0.792 & | 3.824 ±0.769 | 0.075 |
| PC 36:1 | 0.451 ± 0.205 | 0.531 ± 0.175 | 0.531 ± 0.174 | 0.092 |
| PC 36:2 | 1.985 ± 0.448 | 2.186 ± 0.383# | 1.890 (0.570-2.410) | 0.066 |
| PC 36:3 | 1.160 (1.025-1.310) # | 1.232 ± 0.245 # | 0.950 (0.570-1.170) | 0.001 |
| PC 36:4 | 1.890 (1.565-2.115) # | 1.659 ± 0.348 #& | 1.270 (0.570-1.535) | < 0.0001 |
| PC 38:3 | 0.455 ± 0.152 | 0.506 ± 0.146 | 0.410 (0.325-0.505) | 0.144 |
| PC 38:4 | 1.240 (1.050-1.415) # | 1.211 ± 0.329 # | 0.790 (0.570-0.980) | < 0.0001 |
| PC 38:5 | 0.440 (0.370-0.490) | 0.437 ± 0.104 | 0.400 (0.320-0.510) | 0.613 |
| PC 38:6 | 0.800 (0.700-0.955) # | 0.605 (0.527-0.735) & | 0.644 ± 0.212 | < 0.0001 |
| SM 34:1 | 1.550 ± 0.341 | 1.449 ± 0.320 | 1.480 ± 0.353 | 0,438 |
| SM 36:1 | 0.333± 0.09 | 0.275 ± 0.080 & | 0.314 ± 0.090 | 0.058 |
| SM 38:1 | 0.370 ± 0.238 | 0.320 ± 0.204 | 0.314 ± 0.096 | 0.490 |
| SM 40:1 | 0.448 ± 0.191 | 0.367 ± 0.182 #& | 0.510 ± 0.212 | 0.057 |
| SM 42:1 | 0.304 ± 0.088 | 0.265 ± 0.730 & | 0.306 ± 0.088 | 0.202 |
| SM 42:2 | 0.956 ± 0.243 # | 0.806 ± 0.238 & | 0.820 ± 0.212 | 0.016 |
| SM 42:3 | 0.433 ± 0.146 # | 0.351 ± 0.145 & | 0.347 ± 0.124 | 0.023 |
| CER 34:1 | 0.030 (0.020-0.030)# | 0.020 (0.020-0.030) | 0.020 (0.012-0.027) | 0.085 |
| CER 40:1 | 0.040 (0.037-0.050) | 0.047 ± 0.011 | 0.040 (0.040-0.040) | 0.208 |
| CER 41:1 | 0.050 (0.030-0.060) | 0.055 ± 0.018 # | 0.043 ± 0.008 | 0.077 |
| CER 42:1 | 0.134 ± 0.046 | 0.137 ± 0.048 | 0.120 (0.100-0.140) | 0.461 |
| CER 42:2 | 0.080 ± 0.026 # | 0.072 ± 0.024 # | 0.055 ± 0.015 | 0.001 |
| CER 43:1 | 0.020 (0.020-0.030) | 0.030 (0.020-0.040) | 0.020 ± 0.004 | 0.033 |

*The table only includes species with a proportion > 0.3%, except for Cer species, which are all included. Data indicate the relative percentage of each lipid specie contained in LDL. Data are expressed as mean and standard deviation (SD) or median (md) and interquartile range (IQR). Differences between 3 groups were assessed using one-way ANOVA or Kruskal-Wallis rank-sum test (p is shown in the right column). A paired t-test or a Wilcoxon matched-pairs signed-rank test (paired data) and a Student’s t-test or the Wilcoxon rank-sum test (unpaired data) were used to compare 2 groups. Significant differences between 2 groups are indicated as # vs Control group and & 1 y vs 7 d (p ≤ 0.05).*

**Supplementary Figures**

1. *
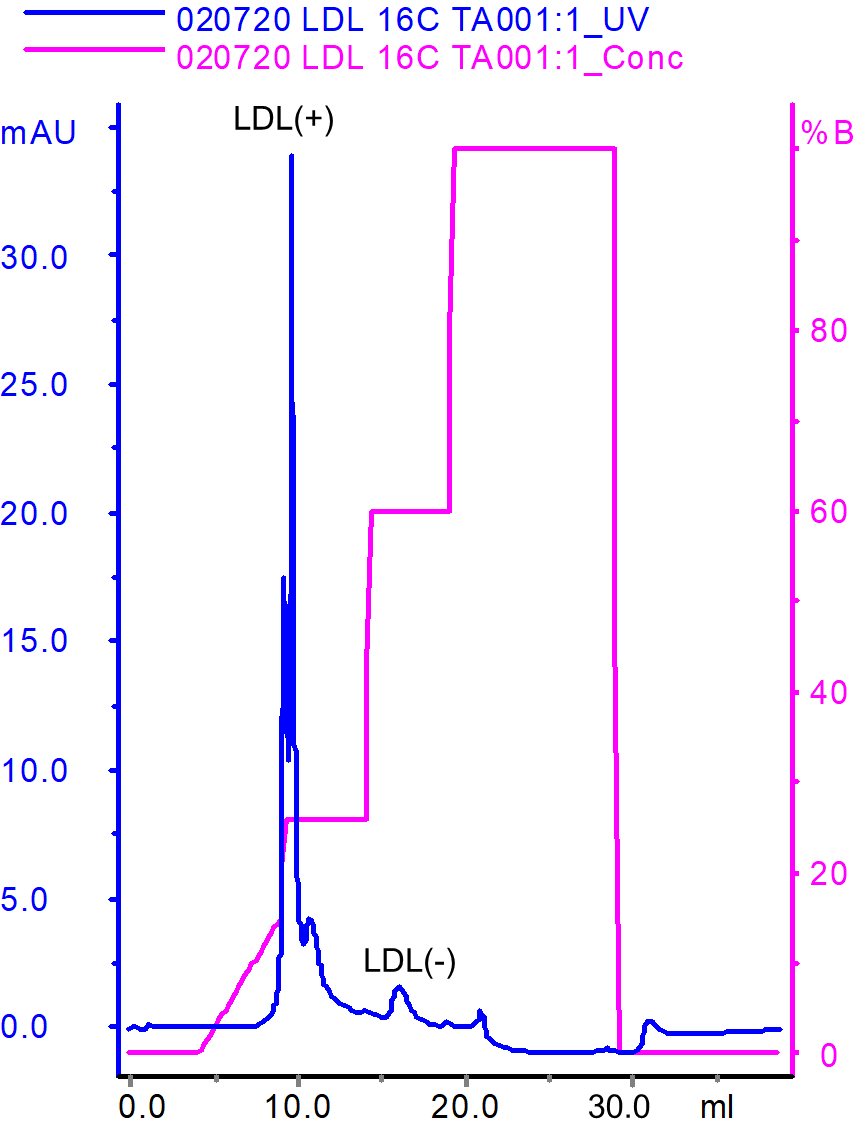
* **B)**

*
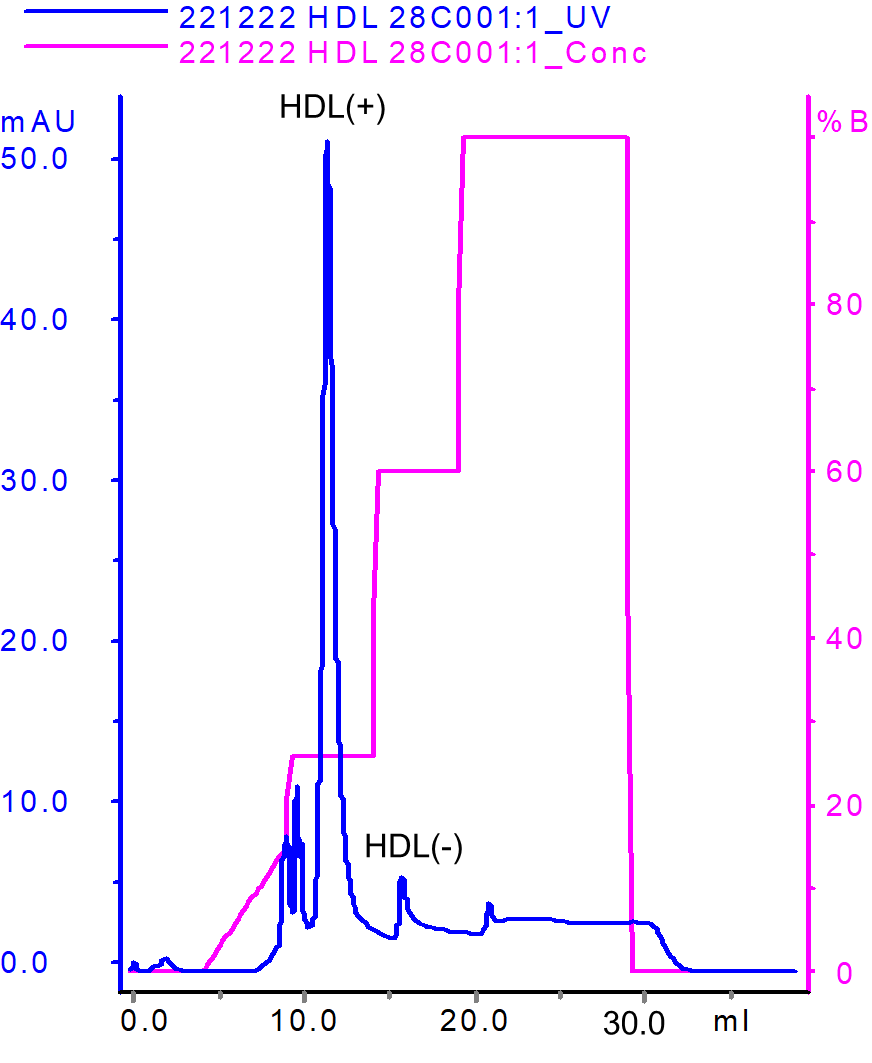
*

*Supplemental Figure S1. Representative chromatogram of LDL(-) and HDL(-) isolation*. Total LDL and HDL (0.2 g apoB/l or apoA-I/l, respectively) were subfractioned by electric charge using anion-exchange chromatography in an AKTA-FPLC system using a MonoQ^TM^ 5/50 GL column and a gradient stepwise method. Electropositive fraction was eluted at 0.26 M NaCl, whereas the electronegative fraction was eluted at 0.6 M NaCl. The proportion of LDL(-) and HDL(-) was calculated from the chromatograms by calculating the area (280 nm peaks) versus total LDL and HDL area, respectively.

*Supplemental Figure S2. VLDL composition. 7 d represents patients 7 days after ischemic stroke; 1 y represents patients one year after ischemic stroke, and Ctr represents the control group. Lipoprotein composition was determined in an autoanalyzer, as described in Methods. A) TC: total cholesterol; B) FC: free cholesterol; C) TAG: triglyacylglycerol; D) PL: phospholipids; E) ApoB; F) ApoE; G) ApoC-III.* *The results are expressed as the percentage of each component in the total mass of the lipoprotein. Data are shown as Tukey box-and-whisker plots. Q1 and Q3 quartiles are the ends of the box, the whiskers extend to 1.5 times de IQR, and dots represent outliers. A paired t-test or a Wilcoxon matched-pairs signed-rank test was used when the samples were paired (7 days and 1 year), whereas the Student’s t-test or the Wilcoxon rank-sum test was used to compare between unpaired samples (7 days vs. controls and 1 year vs. controls). Horizontal bars indicate statistically significant differences between the groups with p ≤ 0.05.*

*Supplemental Figure S3. Cer ratios in LDL. Cer species in LDL were quantified by lipidomic analysis, as described in Methods. 7 d represents patients 7 days after ischemic stroke; 1 y represents patients one year after ischemic stroke, and Ctr represents the control group. A) Cer 16:0/Cer 24:0 ratio and B) Cer 24:1/Cer 24:0 ratio; Results are shown as* *Tukey box-and-whisker plots. Q1 and Q3 quartiles are the ends of the box, the whiskers extend to 1.5 times de IQR, and dots represent outliers. A paired t-test or a Wilcoxon matched-pairs signed-rank test was used when the samples were paired (7 days and 1 year), whereas the Student’s t-test or the Wilcoxon rank-sum test was used to compare between unpaired samples (7 days vs. controls and 1 year vs. controls). Horizontal bars indicate statistically significant differences between the groups with p ≤ 0.05.*
